# Profile of Prescription Medication in an Internal Medicine Ward

**DOI:** 10.3390/healthcare9060704

**Published:** 2021-06-10

**Authors:** Carla Perpétuo, Ana I. Plácido, Jorge Aperta, Maria Teresa Herdeiro, Fátima Roque

**Affiliations:** 1 Research Unit for Inland Development, Polytechnic Institute of Guarda (UDI-IPG), 6300-559 Guarda, Portugal; carladicarol@ipg.pt (C.P.); apertajorge@gmail.com (J.A.); 2 Local Health Unit of Guarda, 6300-035 Guarda, Portugal; anaplacido@ipg.pt; 3 Institute of Biomedicine (iBiMED-UA), Department of Medical Sciences, University of Aveiro, 3810-193 Aveiro, Portugal; teresaherdeiro@ua.pt; 4 Health Science Research Center (CICS-UBI), University of Beira Interior, 6201-001 Covilhã, Portugal

**Keywords:** older adults, polypharmacy, internal medicine ward

## Abstract

Aging-related loss of resilience associated with the lack of evidence regarding the therapeutic efficacy of medicines can prompt a lack of efficacy of treatments and multiple prescriptions. This work aims to characterize the medication profile of Portuguese older adult inpatients and explore the relationship between hospitalization days and the consumption of medicines. A retrospective data analysis study in older patients who were admitted to a medical internal medicine ward during 2019. The median age of the 616 patients included was 85 years. During the hospitalized period, patients took on average 18.08 medicines. The most prescribed drugs belong to the subgroup of (a) anti-thrombotic agents (6.7%), with enoxaparin being the most prescribed, (b) other analgesics and antipyretics (6.6%), paracetamol being the most frequent, and (c) the Angiotensin Conversion Enzyme Inhibitor (ACE) (6.5%), captopril being the most frequent. The high number of prescriptions in older adults during their hospitalization suggests the need of changing therapeutics to achieve a better efficacy of treatment, which corroborates the hypothesis that the lack of scientific evidence concerning the risk/benefits of many medical therapies in older adults can make it difficult to achieve good clinical outcomes and promote the wastage of health resources.

## 1. Introduction

In the last century, the development of health technologies and the improvement in socio-economic conditions have enhanced health and improved life expectancy, which in association with the decrease in fertility has contributed to an aging population [1,2]. Aging is characterized by progressive alterations in psychological, biological (with drug pharmacodynamics and pharmacokinetics alterations), and even social functions and greater susceptibility to disease [3]. Such alterations can cause a decrease in the ability to recover from unhealthy conditions and consequently can increase the consumption of health resources, which includes medicines [4,5]. Recently, it was reported that about four out of 10 older adults consume five or more medicines (polypharmacy) [6]. Pharmacotherapy can improve quality of life, cure, prevent, or relieve symptoms, but in the older population, special care must be taken with the occurrence of adverse drug reactions (ADR) [7]. The increased prevalence of ADR in older adults is not only related to aging-related increases in susceptibility but also the lack of scientific evidence concerning the risk/benefits of many medical therapies of the older adults [8]. Across history, older adults have been systematically excluded from clinical trials [9], and even when they were included, they are younger than the mean age of older adults’ population [8]. As a result, sometimes, prescription can occur without adequate clinical data, which can compromise clinical outcomes and the well-being of the patients [8,9].

For this reason, new approaches are needed to improve the therapeutic efficacy of older adults as well as their quality of life. In this context, the knowledge medication profile of older adults is preponderant. This work aims to characterize the medication consumption profile of inpatient older adults, as well as attempt to establish a correlation between the medication profile and the diseases and hospitalization days.

## 2. Materials and Methods

A retrospective study was performed to characterize the medication profile among older inpatients of a general internal medicine service of a first-level hospital located in the inner center region of Portugal. All older patients (aged ≥ 65) hospitalized in the internal medicine service for at least 4 days during 2019 were eligible to participate in the study. Older patients hospitalized for less than 4 days were excluded. For patients hospitalized more than once in the internal medicine service, the number of days hospitalized was obtained through the sum of the days of each hospitalization. Data were retrospectively collected from the hospital’s electronic medical record and included patient age, patient gender (male/female), patient diagnoses, hospitalization days, and drugs prescribed. The list of all medication, extracted from the electronic records, was converted to the corresponding Anatomical Therapeutic Classification (ATC) code, using the WHO Collaborating Centre for Drug Statistics Methodology’s web [10], and patient’s diagnoses were classified according to the International Statistical Classification of Diseases and Related Health Problems, 10th Revision (ICD-10). Statistical and descriptive analysis was conducted using the IBM SPSS software version 25.0 and Microsoft Excel. Spearman’s test was used to examine the relationship between age, gender, hospitalization days, the most prescribed pharmacological subgroups, and the number of simultaneous prescribed medicines. Numerical and ordinal data were analyzed using descriptive statistics and presented in frequency and percentage and using mean, median, and quartile values.

## 3. Results

A total of 616 participants were included in the study (median age = 85.0, Min 65, Max 100). Most of the participants were male (51.84%), and 90.2% had been hospitalized only one time (median of hospitalized days = 12). The most frequent diagnosis of the 616 inpatients in the study were as follows: (a) I00-I99-Diseases of the circulatory system (21.40%, N = 829), (b) E00-E89-Endocrine, nutritional, and metabolic diseases (N = 636, 16.40%), and (c) J00-J99-Diseases of the respiratory system (10.70%, N = 415) (Table 1). During the hospitalized period, patients took a median of 17.0 medicines (Min 5, Max 50), and the median of simultaneous medicines per day was 12 medicines (Min 3, Max 27) (Table 1).

Within the 11,159 prescribed medications, 285 were different medicines, 137 were dietary supplements, and 28 were enteral or parenteral nutrition. The most prescribed medicines belong to the ATC groups blood and blood-forming organs (23.4%), cardiovascular system (20.5%), nervous system (17.1%), and tract alimentary and metabolism (17.0%) (Appendix A, Table A1). The most prescribed drugs belong to the subgroup of (a) anti-thrombotic agents (6.7%), with enoxaparin being the most prescribed, (b) other analgesics and antipyretics (6.6%), paracetamol being the most frequent, (c) the Angiotensin Conversion Enzyme Inhibitor (ACE) (6.5%), captopril being the most frequent, and (e) irrigation solutions (6.3%), with sodium chloride solutions being the most used (Table 2).

We observed a positive correlation between the hospitalization days and the ICD-10 diagnosis: R00-R99—Symptoms, signs, and abnormal clinical and laboratory findings, not elsewhere classified (R = 0.103, *p* = 0.010) and S00-T88—Injury, poisoning, and certain other consequences of external causes (R = 0.106, *p* = 0.009) (Table 3).

A negative association between age and the medicines belonging to the subgroups A10A (R = −0.111, *p* = 0.006) and N05B (R = −0.110, *p* = 0.006). It was also observed a positive association between age and the medicines belonging to the subgroups B05C (R = 0.165, *p* < 0.0001), C03C (R = 0.171, *p* < 0.0001), J01C (R = 0.119, *p* = 0.003) and R03A (R = 0.106 and *p* = 0.009) (Table 4).

We also observed a positive correlation between the number of hospitalization days and the number of simultaneous prescribed medicines per day (Table 5).

## 4. Discussion

This study analyzed the medication profile of Portuguese inpatients at an internal medicine service and concluded that during hospitalization, the inpatients consumed a high number of medicines, suggesting that the high frailly of older adults associated with the lack of prescription guidelines for older adults made it difficult to achieve clinical outcomes and increased the time of hospitalization.

The high average age of the participants included in this study is not surprising, since according to Eurostat, Portuguese have an average life expectancy of 81.5, which is higher than the mean of 27 European Union countries (81.0). However, the increase in life expectancy is not accompanied by health quality; indeed, only 9% of Portuguese older adults are considered healthy, which is a lower number when compared with Austria (58.0%), Germany (38.0%), and France (37%) [11]. This unhealthy state and aging-related loss of resilience and pharmacokinetic and pharmacodynamics alterations that occur in older adults [12] can be a major contribution to the high average number of hospitalized days [13] as well as to the fact that almost 10% of the participants had more than one hospitalization during 2019.

On average, the participants consumed 18.08 medicines during their hospitalization, suggesting a high complexity of the therapeutic treatment that perhaps results from the multiple comorbidities presented by the participants. Similar results were observed by other studies in a long-term care hospitalization setting [14]. There is a lack of evidence for the use of certain medicines in older adults, which greatly limits knowledge about the effectiveness of medication [15] in this age group and leads to the need for a frequent change in medication. The drugs that act on the nervous system are one of the most frequently prescribed drugs among our patients [16]. Indeed, according to the literature, the consumption of these medicines is frequent not only in hospitalized patients but also in nursing home residents [17,18,19]. In our study, we observed a decrease in the consumed anxiolytics with aging, suggesting an attempt to deprescribe it with increasing ages [5,20,21].

Although the relevant information is provided, the data of this study are not representative of all populations, and they cannot be generalized to all hospitalized older adults; the information collected in this study reinforces the need for more scientific knowledge concerning the risk/benefits of polypharmacy in older adults.

## 5. Conclusions

The association between a high number of prescribed medicines and the number of hospitalization days observed suggests the need for more scientific evidence regarding therapeutic efficacy in older adults.

## Figures and Tables

**Table 1:** Study population characteristics.

Study Population Characteristics	N = 616
Age (years)	
Median (Q1–Q3)	85.0 (78.0–89.0)
65–74	98 (15.9%)
75–84	206 (33.4%)
≥85	312 (50.7%)
Gender	
Female	298 (48.4%)
Male	318 (51.6%)
Hospitalization days	
Median (Q1–Q3)	12 (8–20)
Range (minimum and maximum)	4–90
No. of hospitalizations	
1 hospitalization	556 (90.2%)
2 hospitalizations	54 (8.8%)
3 hospitalizations	6 (1.0%)
No. of prescribed medicines	
Median (Q1–Q3)	17 (13–22)
Range (minimum and maximum)	4–50
No. of simultaneous medicines prescribed per day	
Median (Q1–Q3)	12 (10–14)
Range (minimum and maximum)	3–27
ICD-10 diagnostics	N = 3873
A00-B99—Certain infectious and parasitic diseases	96 (2.50%)
C00-D49—Neoplasms	79 (2.00%)
D50-D89—Diseases of the blood and blood-forming organs and certain disorders involving the immune mechanism	220 (5.70%)
E00-E89—Endocrine, nutritional and metabolic diseases	636 (16.40%)
F0-F99—Mental, Behavioral, and Neurodevelopmental disorders	140 (3.60%)
G00-G99—Diseases of the nervous system	82 (2.10%)
H00-H59—Diseases of the eye and adnexa	11 (0.30%)
H60-H95—Diseases of the ear and mastoid process	14 (0.40%)
I00-I99—Diseases of the circulatory system	829(21.40%)
J00-J99—Diseases of the respiratory system	415 (10.70%)
K00-K95—Diseases of the digestive system	125 (3.20%)
L00-L99—Diseases of the skin and subcutaneous tissue	50 (1.30%)
M00-M99—Diseases of the musculoskeletal system and connective tissue	80 (2.10%)
N00-N99—Diseases of the genitourinary system	396 (10.20%)
Q00-Q99—Congenital malformations, deformations, and chromosomal abnormalities	1 (0.00%)
R00-R99—Symptoms, signs, and abnormal clinical and laboratory findings, not elsewhere classified	278 (7.20%)
S00-T88—Injury, poisoning, and certain other consequences of external causes	53 (1.40%)
V00-Y99—External causes of morbidity	32 (0.80%)
Z00-Z99—Factors influencing health status and contact with health services	336 (8.70%)

**Table 2:** Most prescribed medicines, third level, pharmacological subgroup.

Most Prescribed Medicines (3rd Level, Pharmacological Subgroup)	Frequency	% N = 11,159
A02B—Drugs for Peptic Ulcer and Gastro-esophageal Reflux Disease (GORD)	489	4.4%
A06A—Drugs for Constipation	381	3.4%
A10A—Insulins and Analogues	489	4.4%
B01A—Antithrombotic Agents	746	6.7%
B05B—I.V. Solutions (I.V. solutions used in parenteral administration of fluids, electrolytes and nutrients)	385	3.5%
B05C—Irrigating Solutions (products used for bladder irrigation, surgical irrigation, incl. instruments)	707	6.3%
B05X—I.V. Solution Additives (I.V. solution additives are concentrated preparations containing substances used for correcting fluid and electrolyte balance and nutritional status)	377	3.4%
C03C—High-Ceiling Diuretics	437	3.9%
C07A—Beta Blocking Agents	334	3.0%
C09A—ACE Inhibitors	723	6.5%
J01C—Beta-Lactam Antibacterials, Penicillins	356	3.2%
N02B—Other Analgesics and Antipyretics	739	6.6%
N05A—Antipsychotics	320	2.9%
N05B—Anxiolytics	298	2.7%
R03A—Adrenergics, Inhalants	314	2.8%

**Table 3:** Spearman correlation between hospitalization days and ICD-10 diagnosis.

		Coefficient Value	*p* Value
Hospitalization days	R00-R99—Symptoms, signs, and abnormal clinical and laboratory findings, not elsewhere classified	0.103	0.010
	S00-T88—Injury, poisoning, and certain other consequences of external causes	0.106	0.009

**Table 4:** Spearman correlation between age and medicines prescribed (third level, pharmacological subgroup).

		Coefficient Value	*p* Value
Age	A10A—Insulins and Analogues	−0.111	0.006
	N05B—Anxiolytics	−0.110	0.006
	B05C—Irrigating Solutions (products used for bladder irrigation, surgical irrigation, incl. instruments	0.165	<0.0001
	C03C—High-Ceiling Diuretics	0.171	<0.0001
	J01C—Beta-Lactam Antibacterials. Penicillins	0.119	0.003
	R03A—Adrenergics, Inhalants	0.106	0.009

**Table 5:** Spearman correlation between the variables of hospitalization days and simultaneous medication per day.

		Coefficient Value	*p* Value
Hospitalization days	simultaneous medicines per day	0.089	0.045

## Data Availability

Not applicable.

## Appendix A

**Table A1:** Most prescribed medicines.

Anatomical Main Group		Frequency	% N = 11,159
A	Alimentary Tract and Metabolism	1901	17%
B	Blood and Blood Forming Organs	2606	23.4%
C	Cardiovascular System	2283	20.5%
D	Dermatologicals	28	0.3%
G	Genito Urinary System and Sex Hormones	144	1.3%
H	Systemic Hormonal Preparations, excl. Sex Hormones and Insulins	220	2.0%
J	Anti-Infectives for Systemic Use	1043	9.3%
L	Antineoplastic and Immunomodulating Agents	17	0.2%
M	Musculo-Skeletal System	151	1.4%
N	Nervous System	1913	17.1%
P	Antiparasitic Products, Insecticides, and Repellents	2	0%
R	Respiratory System	800	7.2%
S	Sensory Organs	27	0.2%
V	Various	24	0.2%
